# The effectiveness of interventions in reducing economic inactivity for people with long term health conditions and disabilities in the United Kingdom: a systematic review

**DOI:** 10.1186/s12889-025-25708-3

**Published:** 2025-12-30

**Authors:** Catherine Haighton, Jill Wales, Ross Wilkie, Joanne Gray, Paul Crawshaw

**Affiliations:** 1 School of Communities and Education, Northumbria University, Coach Lane Campus, Newcastle upon Tyne, NE7 7XA UK; 2 School of Social Sciences, Teesside University, Humanities & Law, Clarendon Building, Middlesbrough, TS1 3BX UK; 3 School of Healthcare and Nursing Sciences, Northumbria University, Coach Lane Campus, Newcastle upon Tyne, NE7 7XA UK; 4 Primary Care Centre Versus Arthritis, School of Medicine, Keele University, Staffordshire, ST5 5BG UK

**Keywords:** Chronic disease, Disabled persons, Economic inactivity, Systematic review

## Abstract

**Background:**

Economic inactivity, the proportion of people aged between 16 and 64 years who are not in the labour force, has increased significantly since 2020. Long-term sickness is the most common reason for economic inactivity. In the North of England, economic activity is lower, and levels of poor health in the working age population are higher, than national averages. The international literature reports many interventions designed to reduce economic inactivity including those designed to ameliorate the impact of health conditions on work participation, however, there is a need for high-quality evidence of what works for people with health conditions living in the United Kingdom (UK), and in particular for those living in areas of high deprivation such as the North of England.

**Methods:**

We conducted a systematic review preregistered with the open science framework. We searched AMED, ASSIA, Business Source Premier, CINAHL, Cochrane Library, MEDLINE, ProQuest Dissertations & Theses Global, PsycARTICLES, Science Direct, and Scopus to November 2023. Eligibility criteria for selecting studies consisted of experimental and observational studies published or in grey literature that examined the efficacy or effectiveness of interventions in reducing economic inactivity in people with health conditions living in the UK. Two reviewers independently screened abstracts and full texts followed by data extraction and synthesis using the Narrative Synthesis Framework.

**Results:**

Twenty-seven reports detailing sixteen unique studies and eight different interventions met the eligibility criteria and were included in the review. There was conflicting evidence for the effectiveness of sanctions on economic inactivity and the most robust evidence for classic individual placement and support although more evidence is needed for time-limited individual placement and support. Included studies highlighted the importance of providing specialist employment advice via skilled and experienced advisors but conflicting evidence on combining employment advice with specific psychological therapies.

**Conclusions:**

Overall, we found limited robust evidence of what works in reducing health related economic inactivity, particularly for populations experiencing elevated levels of socio-economic deprivation.

**Open science framework preregistration:**

https://osf.io/aucz9.

**Supplementary Information:**

The online version contains supplementary material available at 10.1186/s12889-025-25708-3.

## Background

Economic inactivity describes the proportion of people aged between 16 and 64 years who are not in the labour force [1]. It includes the retired (specifically those who have taken retirement before the age of 64), students, homemakers, those considered long-term sick due to long-term health conditions or disabilities, people who may have given up looking for work as well as those who choose not to work, perhaps because they have access to adequate financial means. The complex and bidirectional relationship between economic inactivity and health is well documented [2]. Those in employment (employed and self-employed) have better health than economically inactive groups, regardless of age, gender, or social class [3]. Low participation in the labour market is associated with poorer health, and the incidence of long-term health conditions and disabilities is associated with higher rates of economic inactivity [4]. People with long-term health conditions may face barriers to employment, including discrimination, failure to implement reasonable adjustments and accommodations, limited access to support services and wider societal challenges (e.g., poor transport infrastructure for travel to work) [5].

Challenges to reducing economic inactivity have been exacerbated by the COVID-19 pandemic [6]. In December 2023 to February 2024, economic inactivity increased to its highest level since 2012 with 9.34 million people aged 16 to 64 inactive (21.7% of the population) [7], an increase of 275,000 people from the same time in the previous year, and an increase of 850,000 people from before the start of the COVID-19 pandemic [8]. This includes over 30,000 people aged 50–69 years, who are at greater risk of never returning to work [9]. The main reason cited for inactivity was self-reported ill health. Long-term sickness accounted for 28% of total inactivity at the end of January 2023, up from 23% at the start of 2019, making it the most common reason for economic inactivity [10]. Simultaneously, there is growing evidence that transitioning from short to long-term sickness is a significant determinant of non-participation in the labour market, with these effects exacerbated by a growing crisis in the UK NHS that has increased waiting times and delayed treatment [11]. The Darzi report on the state of the NHS in 2024 [12] reiterated that the condition of the UK’s health had deteriorated, driven by factors such as long term health conditions and exacerbated by the critical condition of the NHS, leading to increases in economic inactivity.

The burden of major illness is projected to increase substantially in England by 2040 (37%), placing significant pressure on the working age population both to fund and care for a high need group [13]. Problematically, the working age population will grow at a much slower rate (4%) over this period, whilst health problems simultaneously increase within this cohort. The latter has significant implications for incidence of economic inactivity. It is clear that economic inactivity is both a significant determinant and a symptom of inequalities in health experienced by populations and is exacerbated in places characterised by socioeconomic deprivation [14].

In the North of England, economic activity is lower and levels of poor health in the working age population are higher than national averages. Despite continued investment from a range of sources, including central government and European funds, levels of economic inactivity in the North of England have remained stubbornly high: 24.9% in 2023; 23.7% in 2015 and 26.4% in 1995 compared to the England average of 21% [1]. The international literature reports many interventions designed to reduce economic inactivity including those designed to ameliorate the impact of health conditions on work participation [15]. In particular, Individual Placement and Support (IPS), which uses a number of strategies to support individuals into employment, looks to have some promise [16–25]. However, evidence for IPS mainly comes from the USA [16–25] and IPS is typically only provided in mental health settings for individuals with severe mental illness. In addition, many evaluations of these interventions in the UK rely on uncontrolled study designs and therefore previous systematic reviews of their effectiveness find it difficult to determine if improved employment chances are due to the interventions themselves or to external factors [26, 27].

Recently the UK Department for Works and Pensions (DWP) announced plans to drive economic growth by tackling economic inactivity across every region of the UK [28]. Consequently, there is a need for high-quality evidence of what works for people with health conditions living in the UK, in particular for those living in areas of high deprivation and in the North of England. Therefore, we aimed to systematically review the evidence for effectiveness of interventions designed to reduce health related economic inactivity in the UK including but not limited to areas of social deprivation and the North of England.

## Methods

### Design

We conducted a systematic review to identify experimental and observational studies that examined the efficacy or effectiveness of interventions in reducing economic inactivity in people with health conditions living in the UK including but not limited to areas of deprivation and in the North of England. We searched both peer reviewed and grey literature, preregistered the review protocol with the open science framework (https://osf.io/aucz9), and have reported the review according to the Preferred Reporting Items for Systematic reviews and Meta-Analyses (PRISMA) (https://www.prisma-statement.org/) [29]. We assert that all procedures contributing to this work comply with the ethical standards of the Helsinki Declaration of 1975, as revised in 2008. Ethical approval for this systematic review was not required, since all data came from publicly available sources.

### Protocol deviations

While we originally planned to complete a scoping review, it became clear during write up that the focused nature of our question was more suited to systematic reviewing methodology therefore we revised our approach and included a risk of bias assessment, details of which were not included in our original protocol. In addition, Electronic Theses Online Service was not available when we undertook searches and therefore, we had to replace it with ProQuest Dissertations & Theses Global and again this deviated from our original protocol.

### Inclusion/Exclusion criteria

Inclusion and exclusion criteria followed the PICO(C)(S) criteria (participants, interventions, comparators, outcomes, context and study design). We included people aged between 16 and 64 years, both male and female who were not in the labour force due to long-term health conditions or disabilities (and/or their carers) living in the UK. Job seekers, working people who were on sick leave, retired people (aged over 64 years) or people who may have given up looking for work (not associated with health conditions or disabilities), students, and homemakers were excluded. We included both return to work initiatives which focused on individuals (e.g. monetary incentives for entering employment or developing skills to enhance employment prospects including, but not limited to, national UK government interventions) and return to work initiatives which focused on the employment environment (e.g. behaviour change interventions directed at employers or work environments). Medical or pharmacological interventions and interventions focused on sheltered employment without transition into the open labour market were excluded.

We only included studies with a comparator group. We included outcomes related to return to work, employment status, employment in the open labour market measured via objective (medical records, insurance databases and/or employment records) and self-reported measures while sickness absence among people in employment was an exclusion criterion. We included studies published or in the grey literature of observational or experimental design including pilot trials where they measured efficacy or effectiveness. Single cross-sectional studies, qualitative studies, opinion pieces, editorials, systematic reviews (once reference lists were checked) and abstracts were excluded. We included studies if carried out in community settings in the UK and written in the English language from 1993 onwards as this date signified the introduction of significant and recurrent investment in place-based initiatives via UK government and European funding, including City Challenge, Single Regeneration Budget, New Deal for Communities, European Social Fund and European Regional Development Fund. Community settings allow for a more holistic approach to addressing issues by considering the social, cultural, and environmental factors that influence individuals within their communities, leading to more sustainable and impactful interventions, particularly when aiming to promote health, well-being, and positive change.

### Search strategy

We used a broad search strategy, developed by the lead author (CH) who is experienced in evidence syntheses/information science and reviewed by co-authors and key stakeholders, to maximise the likelihood of identifying all relevant studies. We searched AMED, Business Source Premier and CINAHL via EBSCO; ASSIA, MEDLINE, PsycARTICLES and ProQuest Dissertations & Theses Global via ProQuest; Cochrane Library; Science Direct, and Scopus between 30/10/23 and 08/11/23.

We also sourced grey literature from authors and wider stakeholders (including the North East Work and Health Group, Tees Valley Labour Market Economic Group and the North East Combined Authority) via a call for evidence and UK organisational websites including: Centre for Analysis of Social Exclusion, Centre for Disability Studies, Centre for Mental Health, Chartered Institute of Personnel and Development, Department of Health and Social Care, Department for Work and Pensions, Disability Rights Commission, Disability Rights UK, HM Revenue and Customs, Institute of Employment Studies, Joseph Rowntree Foundation, National Institute of Economic and Social Research, Royal National Institute for Deaf People, Royal National Institute of Blind People, Scottish Government, Social Firms UK, Social Policy Research Unit, Strathclyde Disability Research Group, and Welsh Parliament (see Supplementary Material 1 for further details of grey literature search).

Search terms including truncations, wildcards, and limits as appropriate are outlined in Supplementary Material 2. Keywords were searched in title and abstract. Searches were limited to English language, and humans from 1993 onwards. Keywords focused on population, intervention, outcome, and study design. We also undertook forwards and backwards citation and reference list searching to identify other relevant studies.

### Study selection

We imported references into Rayyan software to manage the screening process across multiple locations. After removal of duplicates, eligibility screening was conducted by two reviewers independently first on title and abstract followed by full text (reviewer one: CH, reviewer two: PC, JG, RW or JW). Disagreements were resolved by discussion. See Supplementary Material 3 for excluded studies at full text screening with reason for exclusion.

### Risk of bias

Risk of bias was assessed in accordance with Cochrane recommendations [30, 31]. Randomised controlled trials (RCTs) were assessed using the Cochrane Risk Of Bias tool version 2 (ROB-2) [32]. Non-randomised studies were assessed using the Risk Of Bias In Non-randomised Studies - of Interventions Version 2 (ROBINS-I V2) tool [33]. Risk of bias assessment figures were created using the robvis tool [34]. Reviewer one (CH) assessed risk of bias for each study which was checked by a second reviewer (PC, JG, RW, or JW).

### Data extraction

We conducted data extraction using a form developed by the lead author (CH) and piloted with one study. Reviewer one (CH) extracted data including details of study aim, population, intervention, study design, outcome measures, results and conclusions. Data extraction was checked by a second reviewer (PC, JG, RW, or JW).

### Data synthesis

For data synthesis we used the Narrative Synthesis Framework [35] to develop the preliminary synthesis, explore relationships between studies and interventions and assess robustness of the conclusions.

### Stakeholder involvement

In addition to the provision of grey literature and review of our search strategy, wider stakeholders were also involved in interpretation of findings. A meeting was held in June 2024 at a local charity providing opportunities for learning, careers advice, access to technology, volunteering opportunities and support. Wider stakeholders represented three different local housing associations that empower people to re-enter the job market, three different local council employment services, citizens advice, a local charity focused on reducing health inequalities and improving the health and wellbeing of people in the local community, an organisation providing employment and skills training, and the local charity who hosted the meeting. Preliminary findings were presented to stakeholders followed by discussion to confirm interpretation of findings.

### Patient and public involvement

While there was considerable stakeholder engagement in the review there was no specific patient and public involvement due to time and resource constraints.

## Results

**Fig. 1:**
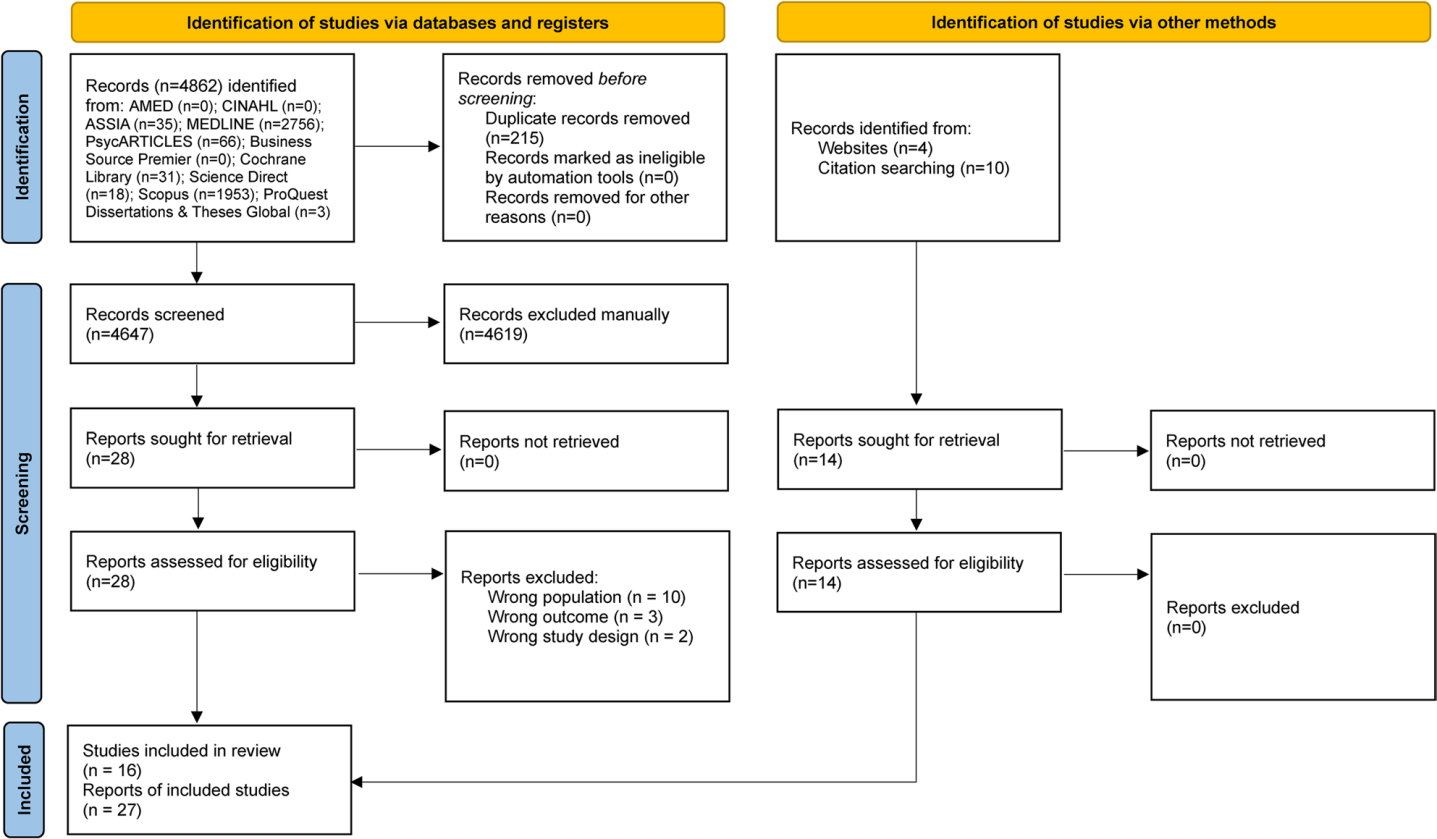
PRISMA flow chart [29]

We included 27 reports detailing 16 unique studies involving 632,821 participants which met the eligibility criteria [36–62] (see Fig. 1). A summary table of included studies is provided in Supplementary Material 4. These reports evaluated Pathways to Work [36, 37], ONE Advisory Service [46, 49, 50], New Deal for Disabled People [51, 57], Individual Placement and Support (IPS) [38–43, 45, 47, 48, 62], IPS delivered by an Employment Specialist [52, 59, 60], IPS LITE [44, 53–56], IPS enhanced with work-focused CBT [61], and Employment Advisers (EAs) in Improving Access to Psychological Therapies (IAPT) [58]. Most studies focused on patients with severe mental illness (*n* = 7) [38–43, 45, 47, 48, 52, 59–62], mild to moderate mental health conditions (*n* = 2) [44, 58] or mild-to-moderate mental and physical health conditions (*n* = 1) [53–56]. Six studies focused on incapacity/disability benefits claimants [36, 37, 46, 49–51, 57]. Studies included five individual RCTs [38–44, 47, 48, 53–56, 61] and eleven non-randomised studies [36, 37, 45, 46, 49–52, 57–60, 62] spanning more than two decades (2001–2023). Studies were carried out in a variety of locations throughout the UK, most commonly in London [38–43, 47, 48, 59, 60] followed by West Midlands [52–56] and Sheffield [53–56]. Other locations included Oxford [44], Glasgow [45], Southampton and Dorset [58], Nottinghamshire [61], and Sussex [62]. Three studies included a number of pilot sites [36, 37, 46, 49–51] incorporating sites in the North of England (Bolton [51], Calderdale and Kirklees [46, 49, 50], East Lancashire [36, 37], Gateshead [36, 37], Leeds [46, 49, 50], North Cheshire [46, 49, 50], North Yorkshire [51] and South Tyneside [36, 37, 51]). One study evaluated a national intervention [57]. In addition, only one study focused specifically on populations experiencing elevated levels of socioeconomic deprivation [47, 48].

### Risk of bias

**Fig. 2:**
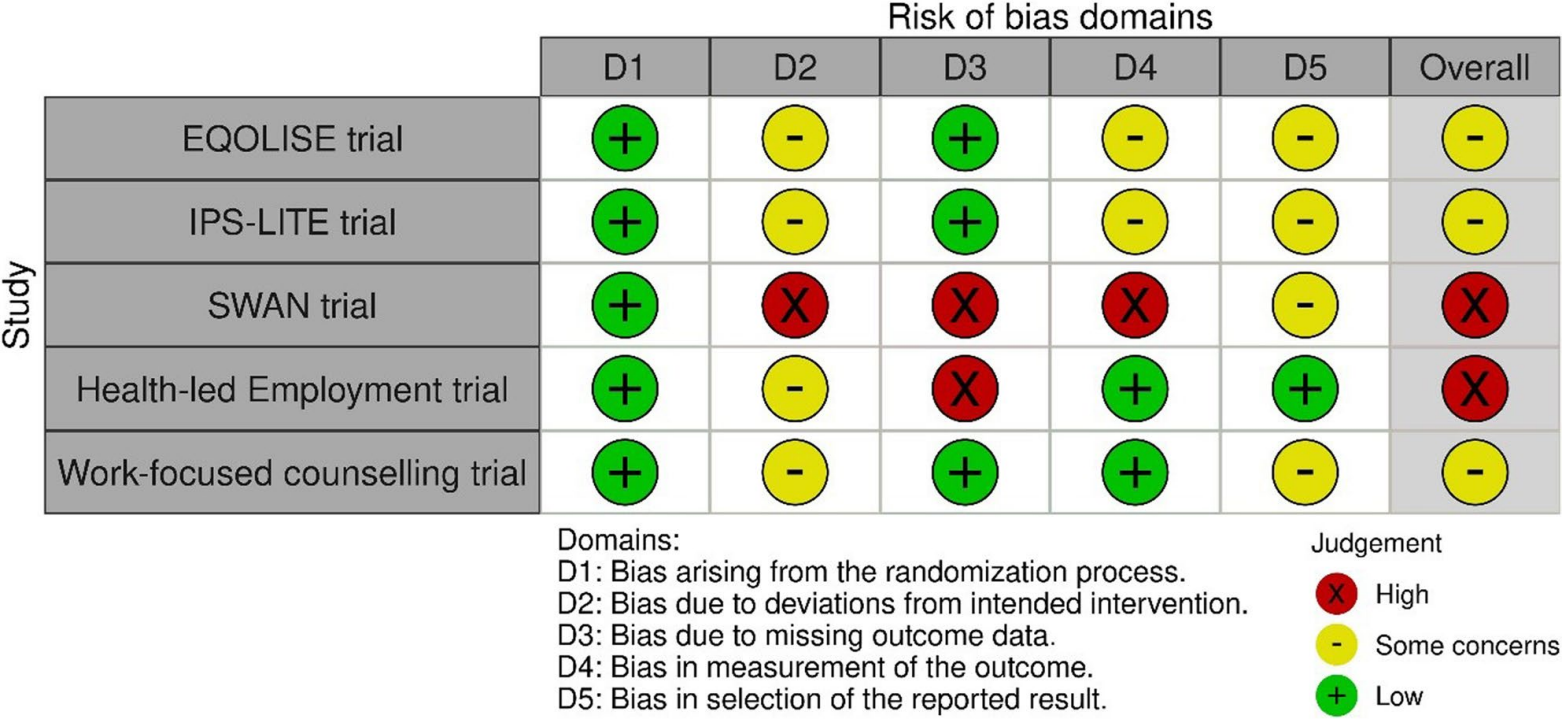
Risk of bias for individual randomised controlled trials

Risk of bias for the five individual RCTs [38–44, 47, 48, 53–56, 61] is shown in Fig. 2. Studies varied on the domains that were most at risk of bias, although all studies showed low risk of bias for the randomisation process, and none were judged as high risk for selection of the reported result.

**Fig. 3:**
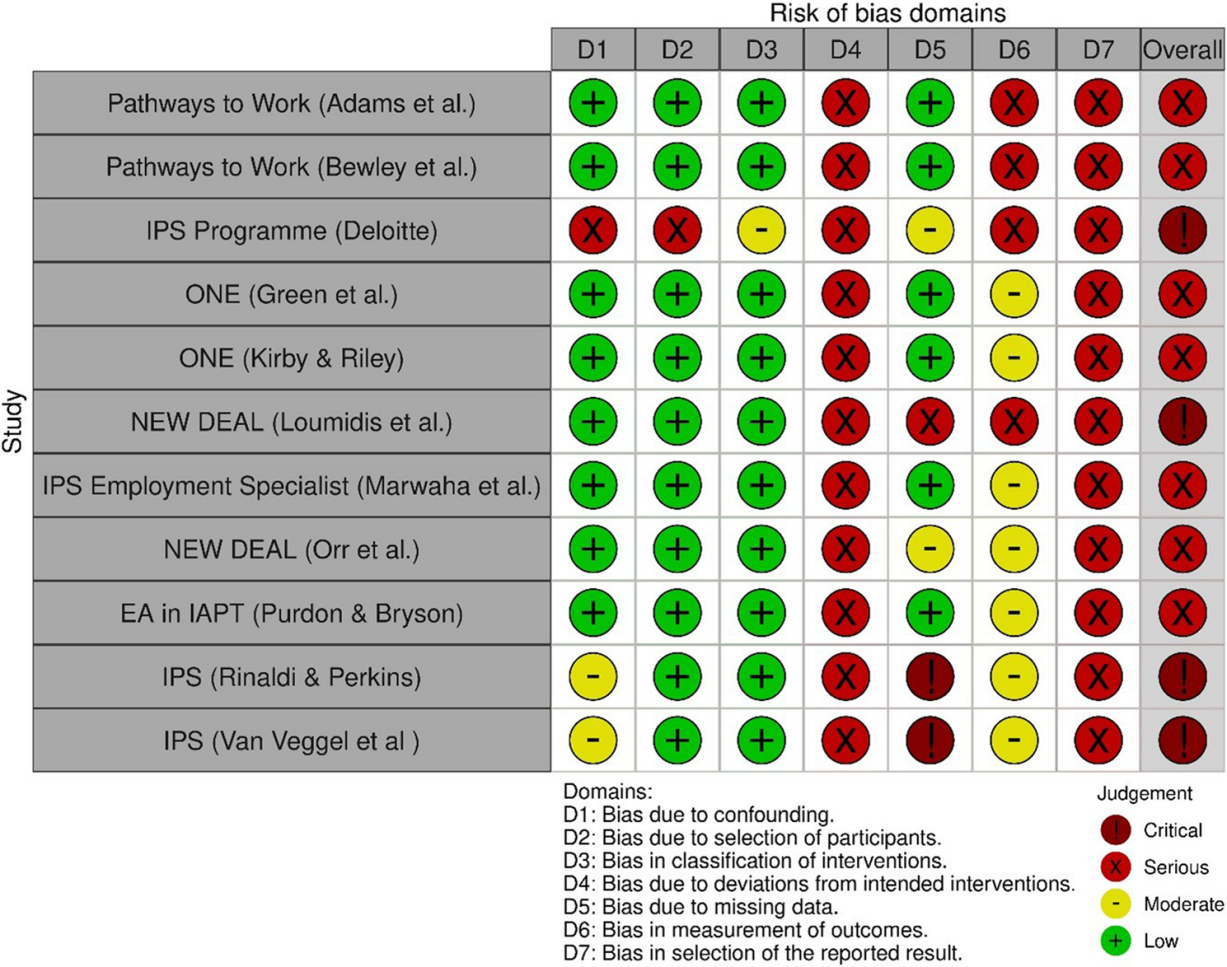
Risk of bias in non-randomised studies of interventions

For the eleven non-randomised studies [36, 37, 45, 46, 49–52, 57–60, 62], risk of bias was often uncertain due to missing information, usually resulting in a serious or critical overall risk of bias (see Fig. 3).

### The interventions

Key elements of each intervention are shown in Table 1. The interventions were grouped according to their key distinguishing features comprising sanctions, classic IPS, time-limited IPS, specialist employment advisors and employment advice with psychological therapies. The effectiveness of interventions with each of these key distinguishing features is discussed.

**Table 1.** Intervention components

**Intervention** **Reference(s)**	**Sanctions**	**Mandatory work focused interview**	**Financial incentives**	**Focus on competitive employment**	**No exclusion criteria**	**Rapid job search**	**Integration with mental health**	**Attention to client’s job preferences**	**Time-unlimited support**	**Benefits counselling**	**Active job development**	**Specialist Employment Advisor**
1. Pathways to work [36]	✓	✓	✓							✓		✓
2. Pathways to work [37]	✓	✓	✓							✓		✓
3. IPS* (EQOLISE) [38–43]				✓	✓	✓	✓	✓	✓	✓	✓	
4. IPS* LITE [44]				✓	✓	✓	✓	✓		✓	✓	
5. IPS* [45]				✓	✓	✓	✓	✓	✓	✓	✓	
6. ONE [46]	✓	✓	✓							✓		
7. IPS* (SWAN) [47, 48]				✓	✓	✓	✓	✓	✓	✓	✓	
8. ONE [49, 50]	✓	✓	✓							✓		
9. NDDP** [51]												✓
10. IPS* [52]				✓	✓	✓	✓	✓	✓	✓	✓	
11. Health-led Employment [53–56]				✓	✓	✓	✓	✓		✓	✓	
12. NDDP** [57]												✓
13. EAs in IAPT*** [58]							✓IAPT†					✓
14. IPS* [59, 60]				✓	✓	✓	✓	✓	✓	✓	✓	✓
15. Work-focused counselling [61]				✓	✓	✓	✓+CBT‡	✓	✓	✓	✓	
16. IPS* [62]				✓	✓	✓	✓	✓	✓	✓	✓	✓

*Individual Placement and Support

**New Deal for Disabled People

***Employment Advisers in Improving Access to Psychological Therapies

†Improving Access to Psychological Therapies

‡Cognitive Behavioural Therapy

### Sanctions

Two interventions, Pathways to Work and ONE Advisory Service, could be clearly distinguished from the others identified through their use of sanctions, the reduction or withdrawal of benefits from claimants on the grounds that they have failed to observe the conditions attached to their benefit claim. Pathways to Work was piloted in 2003/2004, completed national rollout in 2008 and is now closed [36, 37]. Pathways to Work involved compulsory interviews for all (repeat and new) incapacity benefit applicants with a focus on employment [36, 37]. Interviews began at two months into the application and then would repeat at four weekly intervals until the applicant had participated in a total of six work focused interviews [36, 37]. Existing incapacity benefit applicants could participate in Pathways to Work on a voluntary basis [36, 37]. Sanctions, such as a reduction in benefits, could occur for new and repeat incapacity benefit claimants who did not attend [36, 37]. Personalised support and advice were provided by a team of specialists including Disability Employment Advisors, Incapacity Benefit Personal Advisors, and Work Psychologists [36, 37]. This team of specialists were tasked with assisting applicants’ re-entry into competitive employment via management of their health problems (Condition Management Programme), the provision of incentives (Return to Work Credit), and facilitated access to other interventions (New Deal) through the Choices package [36, 37].

ONE Advisory Service was a one stop shop for applicants for welfare benefits including the unemployed and those seeking benefits for long term health conditions and disabilities [46, 49, 50]. ONE Advisory Service was initially introduced in 12 locations (including Calderdale and Kirklees, Leeds and North Cheshire) [46, 49, 50]. In these areas the Benefits Agency and the Employment Service were combined using three different delivery modes: Private/Voluntary Sector, Call Centre, and Basic (1999–2001) [46, 49, 50]. Originally ONE Advisory Service was optional for all (repeat and new) benefits applicants who were provided with a Personal Advisor. Applicants were invited to take part in an interview, with their Personal Advisor, which focused on employment including reviewing employment barriers, readiness and options [46, 49, 50]. The Personal Advisor was also able to provide advice on appropriate benefits (including benefits while employed) and deal with any claims, as well as determine how much better off claimants could be in terms of income and other financial support if they were to return to work or if their circumstances changed [46, 49, 50]. However, sanctions were imposed in April 2000 in these areas requiring those in receipt of benefits to take part in interviews with their personal advisor to discuss return to work [46, 49, 50]. Once again failure to attend these work focused interviews resulted in withdrawal of benefits [46, 49, 50].

In total, four studies evaluated interventions involving sanctions [36, 37, 46, 49, 50]. Two of the studies aimed to evaluate Pathways to Work [36, 37] while two aimed to evaluate ONE Advisory Service [46, 49, 50]. Overall, Pathways to Work proved successful in increasing employment in incapacity benefit claimants [36, 37]. A study using a ‘difference-in-differences’ approach compared outcomes in a cohort of 8035 benefits claimants in seven Pathways to Work pilot areas (including East Lancashire, Gateshead and South Tyneside) and those in carefully chosen comparison areas [36]. At 42 weeks, the Pathways to Work cohort were more likely to be working (+ 9.4% *p* < 0.001), report an increase in income (+£71.73, *p* < 0.001) less likely to be in receipt of incapacity benefit (−8.2%, *p* < 0.001) and less likely to state a health problem affecting employment (−2.9%, *p* < 0.05) [36]. Results did not differ by gender or age [36]. A subsequent study using the same ‘difference-in-differences’ methodology with 5784 benefits claimants in the seven Pathways to Work pilot areas [37] showed the Pathways to Work cohort were more likely to still be working a year and a half later (+ 7.4%, p = 0.09) [37]. Conversely, there were no long-term improvements in income, receipt of incapacity benefit, or health problems affecting employment [37]. Females (13%, p < 0.05) and those who had children to support (17.6%, p < 0.05) were more likely to be employed [37]. However, only new applicants for incapacity benefits were eligible for the Pathways to Work pilots, which limits the degree to which the effects can be generalised, as this represents less than 10% of the total population claiming the benefit [37]. In addition, results revealed that the type of work secured through the Pathways to Work pilots was often part time, limited by participants long term health conditions, disabilities and caring responsibilities [37].

Conversely, the evidence did not suggest that ONE improved rate of employment or reduced receipt of benefits for the economically inactive with long term health conditions and disabilities [46, 49, 50]. One study of 4785 people with long term health conditions and disabilities reported that rates of employment (of two days or more per week) increased for both the cohort accessing the ONE advisory service (24% to 28%) and a controlled comparison group (20% to 25%) however this was not a statistically significant difference [46]. There was, however, a significant difference by delivery model with the basic delivery model outperforming the call centre and private-voluntary sector models although with no clear reason why [46]. A second study, again using a difference in difference design, of a random cohort of 29,451 benefits claimants in the UK reported no difference in the receipt of benefits following the ONE Advisory Service compared to a comparison group [49, 50]. The study did find however that claimants left benefits more quickly when involved in the earliest phases of the ONE Advisory Service compared to those involved in the later phases suggesting a decline in employment outcomes and a potential cohort effect [49, 50]. The evidence did not suggest that the ONE Advisory Service (via any of the models of delivery) was effective in increasing the numbers of people with long term health conditions and disabilities leaving benefits [49, 50]. There was little evidence to suggest that the ONE Advisory Service improved employment in the economic inactive although the results were based on only a 5% sample of all eligible benefits claimants in the UK [49, 50].

### Classic individual placement and support (IPS)

Individual placement and support (IPS) is a form of vocational rehabilitation based on eight standards (1) prompt search for employment; (2) emphasis on working in the open job market; (3) based on individual’s employment choices; (4) continued employment progression; (5) advice regarding benefits; (6) no grounds for eligibility; (7) no time restrictions; (8) situated with mental health teams [38–43, 45, 47, 48, 62]. Four studies evaluated effectiveness or cost effectiveness of IPS [38–43, 45, 47, 48, 62]. All but one study found IPS to be effective and cost effective. One study, which found no effect at one year and a very small significant effect at two years, was based in an area of socioeconomic deprivation and used a conservative definition of employment (30 days or more in a job, rather than the usual 1 day) [47, 48].

The European trial to improve quality of life in severe mental illness with supported employment (EQOLISE) study, was a multi-site randomised controlled trial (RCT) across six European centres [38–43] with one centre in London (*n* = 50). In this trial site 48% of participants commenced employment over 18 months of assistance via IPS, compared to 16% in the control group of existing vocational services [38–43]. IPS was significantly more effective than the existing vocational service in terms of vocational outcomes (difference 32.0%, 95% CI 7.7–56.3) [38–43]. There were, however, considerable differences in findings across the six European centres in terms of IPS effectiveness, thought to be accounted for by differences in labour market conditions in each area [38–43]. Employment also improved social and clinical functioning particularly in the group accessing existing vocational services which could suggest that those patients with the most severe long term health conditions were helped by IPS rather than existing vocational services [38–43]. Findings also suggest that IPS had positive effects on both clinical and employment outcomes in patients with schizophrenia [38–43]. Overall IPS was reported as being more cost effective, in terms of health and social care, than existing vocational services [38–43]. Again, it is interesting to note that the types of work secured through IPS were typically low skilled or support roles even in favourable labour market conditions [38–43]. In addition, recruitment to the study was especially difficult in two countries with a substantial benefit trap (the UK and Netherlands), which occurs when tax and welfare systems make it financially unattractive for individuals to work, as extra earnings can lead to a loss of benefits or payment of higher taxes, resulting in little or no net gain or potentially a loss of income [38–43].

Another study assessed the economic impact of IPS versus traditional vocational schemes for service users, the Scottish government and the NHS [45]. Data was based on individuals with severe and enduring mental health conditions using an IPS service in Glasgow in 2016 (*n* = 126 of which 41 secured employment) [45]. Authors reported that IPS had an estimated net economic impact of £180,970 for the year 2016 or £1,400 per user and concluded that because more users secure employment via IPS and it was cheaper than alternative traditional vocational schemes it had a positive economic impact [45]. However, these findings were based on several assumptions from the literature [45].

The Supported Work and Needs (SWAN) study was a pragmatic RCT which aimed to investigate the effectiveness and cost-effectiveness of IPS in patients with severe mental illness at one and two years in two distinct areas of London [47, 48]. There was no significant difference between groups after one year with only 13% of patients receiving IPS finding work compared to 7% of patients receiving existing services [47, 48]. There were also no significant differences in period of employment, income or hours employed each week [47, 48]. However, a third of patients in the IPS group failed to engage with the employment advisor [47, 48]. The average number of sessions undertaken with the employment advisor was 14 [47, 48]. 86% of patients were followed up after 24 months, but results revealed few were working in the competitive labour market across both arms of the trial [47, 48]. However, significantly more patients (*P* = 0.041) were employed in the IPS arm (22%) compared to the control arm (11%) [47, 48]. Costs did not significantly differ by arm [47, 48]. The study context of an area of socioeconomic deprivation and an intervention provided by an external agency may have made implementation more difficult [47, 48]. However, these unexpectedly low results are partly explained by a conservative definition of employment, 30 days or more in a job, rather than the usual 1 day or more of competitive employment [47, 48].

Finally, in a between group study, results were compared pre (*n* = 140) and post (*n* = 107) IPS across three mental health services which implemented IPS (*n* = 446) [62]. Findings revealed IPS led to significantly more patients (24.9%) working in the open labour market compared to before IPS (14.3%) [62]. IPS also resulted in employment of a greater number of hours per week with 24.3 h/week compared to 15.4 h/week and a shorter time to securing employment with 153 days to employment compared to 371 days [62]. Authors note however, a vulnerability of IPS to loss of effect size over wider reaching implementation due to issues attaining required levels of work in a competitive labour market and maintaining fidelity of intervention [62].

### Time-limited individual placement and support (IPS)

The term IPS-LITE has been used to refer to IPS where support is time-limited (in standard IPS it is not) [44, 53–56]. Typically, IPS-LITE allows approximately 40 weeks for employment searching followed by 18 weeks of support during employment [44, 53–56]. Two studies aimed to evaluate this time-limited IPS [44, 53–56]. One study reported IPS-LITE may be more cost-effective than IPS [44] although the other study reported no evidence of an employment or earnings effect of IPS LITE but there were small impacts on health and wellbeing both statistically significant at the 95% level [53–56]. An RCT of IPS (*n* = 61) compared to IPS-LITE (*n* = 62) [44] found similar numbers of IPS-LITE patients (41%) finding work after a year and a half compared to standard IPS (46%) although 97% of IPS-LITE patients had been released from the IPS-LITE service compared to only 28% from standard IPS [44]. At 40 weeks only 6% of IPS-LITE patients and 11% of standard IPS patients were in work [44]. There were also no significant differences in social or clinical functioning, duration of work, days employed or length of time to first job [44]. Authors reported that standard IPS and IPS-LITE were as effective as each other and that there was little to be gained by providing employment search services beyond 40 weeks [44]. However, as the IPS-LITE services came to an end more quickly than standard IPS this provided the potential for more patients to make use of the LITE service with authors calculating that the extra capacity matched with the service outcomes could results in an additional 17% of patients finding work at 40 weeks compared to standard IPS [44]. Therefore, authors suggested that IPS-LITE was a more cost-effective alternative to standard IPS [44].

The Health-led Employment RCT [53–56] compared IPS-LITE to a control group in people with long term health conditions and disabilities who were economically inactive in West Midlands Combined Authority (WMCA) and Sheffield City Region (SCR) (*n* = 7,266). In WMCA, IPS-LITE had a significant effect on the likelihood of working for more than three months across the 12 months after being randomised (4% points at the 99% significance level) with the IPS-LITE group being 20% more likely, than the control group, to have worked for more than three months after being randomised [53–56]. While there were no effects on health and wellbeing in the WMCA IPS-LITE group, there were improvements in the SCR IPS-LITE group [53–56]. When both WMCA and SCR groups were pooled there was no evidence of an employment or earnings effect of IPS-LITE but there were small effects on health and wellbeing both statistically significant at the 95% level [53–56]. A cost benefit analysis, based on the effects of IPS-LITE on health and wellbeing outcomes rather than outcomes associated with employment, reported a return on investment for IPS-LITE of £1.22 for every £1 spent across both WMCA and SCR groups [53–56]. Results of the Health-led Employment RCT were influenced by the authors choice of primary outcome measure (working for more than three months) which, while being more realistic of true employment status was more challenging to achieve than other similar studies [53–56].

### Specialist employment advisors

Four studies focused on the importance of providing specialist employment advice via skilled and experienced advisors [51, 52, 57, 59, 60]. Two studies evaluated effectiveness of IPS delivered by an Employment Specialist versus existing staff [52, 59, 60] while two studies evaluated New Deal for Disabled People [51, 57] which provided specialist personal employment advisors. Evidence suggested an increase in exit from benefits and increases in employment [51, 57] and that employment specialists may be more effective than existing staff in providing IPS [52, 59, 60]. Marwaha et al. [52] used a natural experiment to assess the effectiveness of IPS provided by a skilled employment advisor compared to IPS provided by existing staff who had received additional training to provide the intervention [52]. Existing staff were employed by three mental health teams with 39 patients while skilled employment advisors was employed by two other mental health teams with 67 patients in the West Midlands [52]. Only those patients (*n* = 96) not currently working at baseline were analysed [52]. Jobs in the open labour market were secured by 10.3% patients receiving IPS from existing staff compared to 22.8% patients receiving IPS from specialist employment advisors and 17.7% of all patients in receipt of IPS [52]. These rates rose to 25.6% for IPS delivered by existing staff and 35.1% for IPS delivered by specialist employment advisors when patients receiving training were also included, however, none of these differences were significant [52]. Authors concluded that it was possible to implement IPS into existing UK mental health teams to increase rates of competitive employment in a timely manner [52]. However, despite the study showing no significant difference in the method of IPS service delivery on rates of competitive employment, authors also concluded that employing a specialist employment advisor was more effective in securing jobs for patients compared to training existing members of staff to provide IPS in addition to their principal role [52].

Another natural experiment [59, 60] assessed the effect of implementing Specialist Employment Advisor delivered IPS (*n* = 8) versus non-integrated pre-vocational services (*n* = 4) in Community Mental Health Teams showing a significant increased rate of competitive employment or training at 6 and 12 months post IPS [59, 60]. The IPS specialist employment advisors supported 38% of patients into employment or training compared to 12% in the non-integrated pre-vocational services (*p* < 0.001) at 6 months [59, 60]. At 12 months the IPS specialist employment advisors supported 39% patients compared to 10% in the non-integrated pre-vocational services (*p* < 0.001) [60]. Cost of supporting a patient into competitive employment in the non-integrated pre-vocational service was 6.7 times higher than in the IPS service [59]. Authors concluded that the results not only corroborate the implementation of IPS in community mental health teams but also indicate the importance of specialist employment advisors in providing the service [59, 60].

The New Deal for Disabled People was piloted in 1998 with a national extension in 2001 [51, 57]. It is now closed [51, 57]. Once extended nationally, all incapacity-related benefit claimants were eligible to take part in the New Deal for Disabled People on a voluntary basis [51, 57]. The service was delivered through a network of 65 Job Brokers (charity/voluntary, private and public sector organisations bid for contracts) [51, 57]. All incapacity-related benefit claimants were invited to take part in an interview about employment on a voluntary basis which triggered access to other work focused opportunities including training, personalised work searching activities, job advice and ongoing support once employed [51, 57]. The New Deal for Disabled People Personal Adviser Service was part of the wider New Deal for Disabled People programme that also incorporated the Innovative Schemes [51, 57]. The Personal Adviser Service pilot offered one-to-one support and guidance to people with a disability or chronic illness on locating, obtaining and remaining in employment [51, 57]. A study by Loumidis et al. [51] (which included Bolton, North Yorkshire and South Tyneside among their study sites) involved a controlled comparison of (*n* = 2557) participants and non-participants in the Personal Advisor Service in their exits from benefits, and found that 11% of those who participated in the Personal Advisor Service were no longer claiming benefits compared to 7% of those who did not participate over a 24 month follow up period. The study also found that those who participated in the Personal Advisor Service exited benefits more quickly than those who did not, although the probability of exiting from benefits was higher for participants who had been claiming benefits for the shortest period of time (*p* < 0.05) [51]. There were no significant differences in exit from benefits between participants and non-participants of the Personal Advisor Service, in addition, exit from benefit did not automatically translate into competitive employment [51].

In a longitudinal study, a large cohort of disability related benefit claimants registered for the New Deal for Disabled People (*n* = 522,596) were compared to a control group consisting of disability related benefit claimants not registered for the New Deal for Disabled People (*n* = 44,049) [57]. After two years there were significant (*p* < 0.05) increases in rates of competitive employment for both new (+ 7%) and current (+ 11%) New Deal for Disabled People registered benefit claimants particularly for long-term/high-rate claimants and those facing multiple or significant barriers to employment [57]. Over two years there were also significant (*p* < 0.001) exits from benefits for both new (−13%) and current (−16%) New Deal for Disabled People registered benefit claimants particularly for older, more disabled long-term claimants and those facing multiple or significant barriers to employment [57]. However, participation in the New Deal for Disabled People was voluntary and as such the intervention sample for the study was not random which could have introduced selection bias as volunteers may have been more motivated to find work [57].

### Employment advice with psychological therapies

Two studies combined employment advice with specific psychological therapies with contrasting results [58, 61]. One study evaluated IPS enhanced with work-focused cognitive behavioural therapy (CBT) [61] but found no additional benefit of counselling over IPS, while another evaluated Employment Advisers (EAs) in Improving Access to Psychological Therapies (IAPT) [58] and found that seeing an EA in IAPT had a significant impact on mental health and on the likelihood of entering the labour market. A pilot pragmatic RCT assessed whether three to six sessions of Psychologist led CBT, focused on employment, added to IPS (*n* = 37) would be more effective than IPS on its own (*n* = 37) to increase economic activity in people with mental health conditions such as schizophrenia [61]. While a third of participants secured competitive employment there was no significant difference between participants receiving CBT + IPS and participants receiving IPS in terms of mean hours per week (hpw) employed at either six month (CBT + IPS 3.89hpw (SD 10.60) v IPS 3.22hpw (SD 9.53)) or 12 month (CBT + IPS 7.07hpw (SD 14.09) v IPS 3.67hpw (SD 7.80)) follow up [61]. Over the course of the study 43% of participants withdrew, particularly those who were not engaging with the services, affecting the power of the study to determine effectiveness of CBT + IPS over IPS alone [61].

NHS England’s IAPT, now known as NHS Talking Therapies, is based on National Institute for Health and Care Excellence (NICE) evidence-based guidance for treatment of people with depression, anxiety, and other frequently occurring psychological problems and was designed to enhance provision of psychological therapies [58]. The combined EAs in IAPT service was designed to combine IAPT with support and advice regarding employment with the aim of improving psychological health while also improving participants ability to return to, stay in or start a new job [58]. Access to either EAs in IAPT or IAPT alone is entirely voluntary [58]. One study evaluated EAs in IAPT by comparing the outcomes of 6,640 IAPT clients, of whom 11% saw an EA in contrast to an IAPT client group who did not see an EA matched on a number of key characteristics [58]. Results showed that, by the last appointment, IAPT clients who were unemployed at baseline and who saw an EA had significantly better mental health, were significantly less likely to believe that their mental health influenced their prospects of employment, were significantly less likely to be in receipt of disability related benefits, and were significantly more likely to be employed than the matched sample who had not seen an EA [58]. EAs also supported participants in identifying and claiming benefits for which they were eligible but were not claiming at baseline [58]. Finally, the study reported that EAs in IAPT did not have any effect on participants self-reported everyday activities [58].

## Discussion

We have shown that there is limited published, high-quality evidence of what works in reducing health related economic inactivity, particularly within populations experiencing elevated levels of socio-economic deprivation such as those found in the North of England. Studies which were available and eligible for inclusion in this review [36–62] provided insufficient evidence of their effectiveness in reducing health related economic inactivity and focused on individual-level, supply-side interventions with no eligible studies focusing on demand-side interventions. There was the most conclusive evidence for Individual Placement and Support (IPS) with three out of four included studies [38–43, 45, 47, 48, 62] reporting IPS to be effective and cost effective in supporting people with mental health problems back into employment. This is not surprising given results from randomised trials and two meta-analyses [16, 17] have shown the effectiveness of IPS in the USA. Results from randomised trials [18–25] have shown that rates for competitive employment on the open job market for patients using IPS were more than doubled. In addition, considering for a moment, just the highest level of evidence, the five studies which used an individual RCT design [38–44, 47, 48, 53–56, 61] all focused on the effectiveness of IPS in some way. These RCTs reported not only IPS to be an effective intervention for reducing economic inactivity [38–43, 45, 47, 48, 62] but that time limited IPS (IPS_LITE) may be a more cost-effective alternative to IPS [44] with significant benefits to health and wellbeing [53–56]. In addition, there were no extra benefits of adding work focused counselling to IPS [61].

It is important to be aware, however, that employment is dependent on local social factors, macroeconomics and public policy, consequently, differences in both labour markets and welfare systems might reduce the effectiveness of IPS. Outside of the United States, IPS has been shown to be effective in studies in Australia [63], Canada [64], Hong Kong [65], and Switzerland [66]. A previous review which examined the evidence for effectiveness of the IPS model within the UK found a paucity of evidence despite including studies without comparator groups and rating the quality of studies as adequate [67]. Authors of the review concluded that after 6 to 18 months IPS led to increases in the percentage of participants who were either in training or employment although this was only where IPS closely followed the intervention protocol [67]. It is interesting to note that the one study in our review which found no effect of IPS at one year and a very small significant effect at two years was based in an area of socioeconomic deprivation in South London [47, 48]. Our review also provides evidence to suggest that IPS and other similar interventions need to be delivered by an Employment Specialist [51, 52, 57, 59, 60] while more evidence is needed on the effectiveness of time-limited IPS [44, 53–56]. We found conflicting evidence on combining employment advice with specific psychological therapies [58, 61]. While there was no additional benefit of IPS enhanced with work-focused CBT [61] seeing an Employment Adviser in Improving Access to Psychological Therapies had a significant impact on mental health and on the likelihood of entering the labour market [58].

We also found contradictory evidence for those interventions which included sanctions as part of their strategy to reduce health related economic inactivity. Successive governments have increased the use of sanctions, or conditionality, with the intention of increasing employment. Pathways to Work included mandatory work-focused interviews for incapacity benefit claimants with non-attendance resulting in benefit deductions while the ONE Advisory Service required claimants to attend a work-focused meeting as a condition of receiving benefits. While Pathways to Work pilots proved successful in increasing employment in incapacity benefit claimants [36, 37], a final evaluation report stated that there was no statistically significant evidence that the effect of Pathways to Work on competitive employment was greater than zero [68]. ONE did not improve rates of employment or reduce receipt of benefits for the economically inactive with long term health conditions and disabilities [46, 49, 50]. Other studies have suggested that sanctions on benefits do not actually increase employment levels [69]. Reed found that there was no evidence that increased use of sanctions in Jobcentre Plus districts between October 2012 and June 2014 led to decreased unemployment or increased employment [69]. While there is no conclusive evidence that conditionality can achieve the desired goal of increasing employment for benefits claimants, there is other evidence to suggest that imposing strict eligibility criteria and sanctions can actually be harmful to disabled people’s health, activity and financial situation [70]. Research has shown that many people with long term health conditions and disabilities worry about being more active for fear of benefit sanctions being imposed, are aware of sanctions being imposed on others, and report being more likely to be more active if threat of sanctions are removed [70]. According to a summary of existing studies, while the evidence base is limited, it is generally robust and indicates that sanctioning may have little to no positive effects, and potentially negative effects, on employment outcomes for disabled people [71]. Further, studies suggest that sanctions can negatively impact mental health and lead to extreme poverty [71].

In addition, while Pathways to Work pilots proved successful in increasing employment in incapacity benefit claimants the non-statistically significant difference for earnings indicates that these employment opportunities may be in poor quality, low paid jobs. The EQOLISE study [38–43] also reported that IPS workers seemed more able to find jobs for individuals with severe mental illness in unskilled, support positions. It is interesting to note that adverse physical and psychosocial conditions of work, poor pay, insufficient hours, temporary work, insecurity, and the risk of redundancy or job loss can all adversely affect health [72]. Conversely, features commonly associated with good jobs include: adequate pay; protection from physical hazards; job security and skills training with potential for progression; a good work-life balance and the ability for workers to participate in organisational decision-making [72]. Increasing the quantity of jobs without consideration of job quality is likely to exacerbate social and health inequalities and create unequal economic growth [72]. This is particularly important in more economically deprived regions such as the North of England, where a skills deficit exists alongside greater health inequalities [72].

On a positive note, since carrying out this review, we are aware of two further evaluations of the effectiveness of interventions in reducing economic inactivity for people with long term health conditions and disabilities in the UK [73, 74]. Work Choices, for people with disabilities or long-term health conditions, used specialist providers to help them find, keep and progress in employment and replaced the WORKSTEP programme which had not been robustly evaluated [73]. While referrals to Work Choices ended in early 2018 an independent evaluation of its effectiveness has only recently been published [73] reporting participants who joined the programme had a higher employment rate and were less likely to be in receipt of benefits compared to a matched group of non-participants [73]. Work Choice was succeeded by the Work and Health Programme, implemented as an RCT, originally aimed to support disabled people, among others, to secure competitive employment [74]. Analysis of the impact of the programme revealed Work and Health programme participants were significantly more likely “to have done some work” than control participants providing further evidence for the role of dedicated employment advisors [74]. However, neither of these evaluations specifically focused on areas of socioeconomic deprivation [73, 74].

### Implications for policy, practice and research

Regional stakeholders have a long-standing commitment to delivering interventions to reduce economic inactivity and currently offer programmes of interventions, including some of those highlighted in our review (e.g. IPS). These interventions have proliferated in a mixed economy of provision across public, private, and voluntary sectors with varying degrees of success. Therefore, further research needs to focus on high quality, robust evaluations of interventions. More evidence is also needed for time-limited IPS, as this may be a cost-effective alternative to IPS, and the value of combining employment advisors with psychological therapies. Research is particularly needed in areas of socioeconomic deprivation and in the North of England to understand the health determinants of economic inactivity, evaluate existing regional interventions, co-produce new models of good practice and ultimately shape policy at a regional level. Finally, more research is needed to understand why employment opportunities associated with these interventions may be in poor quality, low paid or unskilled jobs.

### Strengths and limitations of the review

We carried out a comprehensive search of both published and grey literature, but as with any systematic review there is a small possibility that we may have missed some potentially eligible literature. No translation services were available however this should not have affected our ability to include all eligible studies as our focus was on the UK. In addition, as outlined in our methods section, we needed to deviate from our original protocol in the following ways. First, Electronic Theses Online Service was not available during the time when we undertook searches and therefore, we had to replace it with ProQuest Dissertations & Theses Global. Second, we originally planned to complete a scoping review, but the focused nature of our question was more suited to systematic reviewing methodology and hence we added a risk of bias assessment. Overall, these deviations resulted in a more robust evaluation of the effectiveness of interventions in reducing economic inactivity for people with long term health conditions and disabilities in theUK.

### Strengths and limitations of the evidence base

This review sought to include only the highest level of evidence of the effectiveness of interventions in reducing economic inactivity for people with long term health conditions and disabilities in the UK, therefore included studies had to be either experimental or observational with a comparator group. Only 16 unique studies met this inclusion criteria, and these were detailed across 27 different reports (both peer reviewed published and grey literature). Overall pooled studies included in excess of 600,000 participants allowing for more robust conclusions to be made. However, only five studies made use of the gold standard individual RCT design with the remaining using non-randomised designs. Risk of bias for the five individual RCTs varied, although all studies showed low risk of bias for the randomisation process. However, for the eleven non-randomised studies, risk of bias was often uncertain due to missing information, usually resulting in a serious or critical overall risk of bias. Finally, there was paucity of evidence in areas of socioeconomic deprivation and in the North of England.

## Conclusions

Health-related economic inactivity continues to be an urgent priority for policymakers. In the UK, the Darzi Report [12], a rapid review of the health of the NHS, highlights both its prevalence and impact on health services and national prosperity. Simultaneously, the UK Department of Work and Pensions is clear in its intention to deliver a comprehensive plan to get people back to work, with the ultimate aim of delivering growth [28]. In the UK and elsewhere, understanding what works in supporting those with long term health conditions back into the labour market has clearly never been more important. Our review has indicated there is limited robust evidence of what works in reducing health related economic inactivity, particularly within populations experiencing elevated levels of socioeconomic deprivation.

## Supplementary Information

Supplementary Material 1.

Supplementary Material 2.

Supplementary Material 3.

Supplementary Material 4.

## Data Availability

All data generated or analysed during this study are included in this published article [and its supplementary information files]. Data used for this review is publicly available.
