# Supplementary material for: The effectiveness of interventions in reducing economic inactivity for people with long term health conditions and disabilities in the United Kingdom: a systematic review
Source: BMC Public Health. 2025 Dec 30;25:4400. doi: 10.1186/s12889-025-25708-3 (PMC12754864; doi:10.1186/s12889-025-25708-3)
Supplement: Supplementary file 1 — Supplementary Material 1. [file 12889_2025_25708_MOESM1_ESM.docx]

Supplementary Material 1: Grey literature search

One of the authors (PC) requested grey literature via a call for evidence from the following groups. Literature was screened by two reviewers independently:

- North East Work and Health Group (at their meeting in September 2023)
- Tees Valley Labour Market Economic Group (via email in September 2023
- North East Combined Authority (via email in September 2023)

The following UK organisational websites were also searched in September 2023 by one of the authors (CH). For those websites that contained a publication page these were either hand searched or searched using the term “economic inactivity” filtered for research, where this was not possible whole websites were searched using the same term. All potentially relevant studies were screened by two reviewers independently:

- Centre for Analysis of Social Exclusion (<https://sticerd.lse.ac.uk/case/>)
- Centre for Disability Studies (<https://disability-studies.leeds.ac.uk/>)
- Centre for Mental Health (<https://www.centreformentalhealth.org.uk/>)
- Chartered Institute of Personnel and Development (<https://www.cipd.org/uk>)
- Department of Health and Social Care (<https://www.gov.uk/government/organisations/department-of-health-and-social-care>)
- Department for Work and Pensions (<https://www.gov.uk/government/organisations/department-for-work-pensions>)
- Disability Rights Commission (<https://www.drc.org.uk/>)
- Disability Rights UK (<https://www.disabilityrightsuk.org/>)
- HM Revenue and Customs (<https://www.gov.uk/government/organisations/hm-revenue-customs>)
- Institute of Employment Studies (<https://www.employment-studies.co.uk/>)
- Joseph Rowntree Foundation (<https://www.jrf.org.uk/>)
- National Institute of Economic and Social Research (<https://niesr.ac.uk/>)
- Royal National Institute for Deaf People (<https://rnid.org.uk/>)
- Royal National Institute of Blind People (<https://www.rnib.org.uk/>)
- Scottish Government (<https://www.gov.scot/>)
- Social Firms UK (<https://socialfirmsuk.co.uk/>)
- Social Policy Research Unit (<https://www.york.ac.uk/business-society/research/spru/>)
- Strathclyde Disability Research Group (<https://www.strath.ac.uk/workwithus/healthwellbeing/researchcentres/disabilityresearchgroup/>)
- Welsh Parliament (<https://senedd.wales/>)
