# Supplementary material for: The effectiveness of interventions in reducing economic inactivity for people with long term health conditions and disabilities in the United Kingdom: a systematic review
Source: BMC Public Health. 2025 Dec 30;25:4400. doi: 10.1186/s12889-025-25708-3 (PMC12754864; doi:10.1186/s12889-025-25708-3)
Supplement: Supplementary file 2 — Supplementary Material 2. [file 12889_2025_25708_MOESM2_ESM.docx]

**Supplementary Material 2 – Full Search Strategy**

tiab(disabled OR disabilit* OR “chronic disease” OR “chronic ill*” OR “chronic absence*” OR “chronic sick*” OR “long standing disease*” OR “long standing ill*” OR “long standing absence*” OR “long standing sick*” OR “longstanding disease*” OR “longstanding ill*” OR “longstanding absence*” OR “longstanding sick*” OR “long term disease*” OR “long term ill*” OR “long term absence*” OR “long term sick*” OR “longterm disease*” OR “longterm ill*” OR “longterm absence*” OR “longterm sick*” OR “long-term disease*” OR “long-term ill*” OR “long-term absence*” OR “long-term sick*” OR “permanent disease” OR “permanent ill*” OR “permanent absence*” OR “permanent sick*” OR musculoskeletal OR “back injur*” or “back damage” OR “back pain” OR “neck injur*” OR “neck damage” OR “neck pain” OR “shoulder injur*” OR “shoulder damage” OR “shoulder pain” OR “disc degeneration” OR “vertebrae damage” OR “upper limb injur*” OR “upper limb damage” OR “upper limb disorder*” OR “upper limb sprain*” OR “muscle injur*” OR “muscle damage” OR “muscle disorder*” OR “muscle sprain*” OR “ligament injur*” OR “ligament damage” OR “ligament disorder*” OR “ligament sprain*” OR “upper extremity disorder*” OR “thoracic pain” OR “calcific tendonitis” OR “shoulder capsulitis” OR “tension neck syndrome” OR “whiplash associated disorder” OR “impingement syndrome” OR tenosynovitis OR peritenonitis OR tendonitis OR epicondylitis OR “carpal tunnel syndrome” OR “repetitive strain injury” OR “upper extremity disorder” OR “upper limb disorder” OR “spinal pathologies” OR “spinal cord injury” OR backache OR “degenerative disc disease” OR “prolapsed disc” OR sciatica Or fibromyalgia OR osteoarthritis OR “inflammatory arthritis” OR “rheumatoid arthritis” OR “ankylosing spondylitis” OR “knee injur*” OR “foot injur*” OR “ankle injur*” OR angina OR “myocardial infarction” OR “abnormal heart rhythm” OR “cardiovascular disease” OR “cardiac failure” OR “arterial disease” OR “coronary heart disease” OR “coronary artery disease” OR hypertension OR “ischaemic heart disease” OR “cardiopulmonary problems” OR “respiratory conditions” OR “occupational asthma” OR “work exacerbated asthma” OR “chronic obstructive pulmonary disease” OR “lung disease” OR “airflow limitation” OR rhinitis OR “mental health” OR dysthymia OR “anxiety disorder*” OR “personality disorder*” OR “panic disorder*” OR “social anxiety” OR phobia OR “post traumatic disorder*” OR “obsessive compulsive disorder*” OR “eating disorder*” OR “affective disorder*” OR “neurological disorder*” OR depress* OR “major depressive disorder” OR stress OR distress OR burnout OR sick OR comorbid* OR cancer)

AND

tiab(rehabilitation OR vocational OR education OR professional OR occupational OR retraining OR “welfare to work” OR “back to work” OR training OR retraining OR re-training OR skill*OR advice OR counse?ling OR “disability insurance” OR “disability benefit*” OR “liability insurance” OR “social security” OR “disability pension” OR “sick leave” OR retirement OR “mobility allowance” OR “disabilit* allowance*” OR “sickness* benefit*” OR “sickness* pension*” OR “premature* retire*” OR “early retire*” OR quota* OR “mobility pension*” OR “invalidity pension*” OR “invalidity allowance*” OR “invalidity benefit*” OR “active labo?r market program*” OR “employ* subsid*” OR “disability living allowance*” OR “attendance allowance” OR “incapacity benefit*” OR “incapacity allowance*” OR “incapacity pension*” OR “severe disablement allowance*” OR “supported work” OR “supported employ*” OR “disabled persons tax allowance*” OR “new deal for disab*” OR “new deal for jobseekers with disab*” OR “access to work program*” OR “pathways to work” OR “work preparation*” OR “job broker*” OR “permitted work rule*” OR “work focus?ed” OR “work-focus?ed*” OR workstep OR work-step OR “disability working allowance*” OR “condition management program*” OR “return to work” OR “work trial*” OR “employment trial*” OR “work placement*” OR “job introduction scheme*” OR “disability discrimination act” OR “new deal innovative scheme*” OR “work preparation” OR “job preparation” OR “new deal personal advis?r*” OR “working tax credit” OR “travel to work” OR “jobseeker* grant*” OR “job seeker* grant*” OR “jobfinder* grant*” OR “job finder* grant*” OR “jobfinder* allowance*” OR “job finder* allowance*” OR “jobseeker* allowance*” OR “job seeker* allowance*” OR “job match” OR workfare OR “work fare” OR “52 week linking rule*” OR “access to work” OR jobmatch OR “biopsychosocial intervention*” OR “psychosocial intervention*” OR “sickness absence*” OR “sickness management” OR “sick pay” OR “early intervention*” OR “work loss” OR “job loss” OR “individual placement and support” OR “employ* advisor*”)

AND

tiab(employ* OR occupation* OR work* OR unemploy* OR vocation* OR labo?r OR job* OR earn* OR paid OR paying OR payment* OR income OR “salaries and fringe benefits” OR salary OR salari* OR wage*)

AND

tiab(“Randomi?ed control* trial*” OR “random* allocate*” OR “random* assign*” OR “double blind” OR “single blind” OR “treble blind” OR “triple blind” OR “clinic* trial*” OR “phase I” OR “phase II” OR “phase III” OR “phase 3” OR “phase IV” OR “phase 4” OR “control* trial*” OR “multicent* study” OR “placebo*” OR randomi* OR crossover OR RCT OR “prospective study” OR quantitative OR observational OR “evaluation stud*” OR “program* evaluation” OR evaluat* OR effective* OR “difference in difference” OR “stepped wedge” OR longitudinal)

AND

mesh.Exact("Employment" OR "Employment, Supported" OR "Clinical Trials as Topic")

Limit Human, English Language, from 01/01/1993, and where possible to UK based studies
