# Supplementary material for: The effectiveness of interventions in reducing economic inactivity for people with long term health conditions and disabilities in the United Kingdom: a systematic review
Source: BMC Public Health. 2025 Dec 30;25:4400. doi: 10.1186/s12889-025-25708-3 (PMC12754864; doi:10.1186/s12889-025-25708-3)
Supplement: Supplementary file 3 — Supplementary Material 3. [file 12889_2025_25708_MOESM3_ESM.docx]

**Supplementary Material 3 - Excluded studies (with reason for exclusion)**

1. Barlow, J. H., Wright, C. C., & Wright, S. (2003). Development of job-seeking ability in people with arthritis: Evaluation of a pilot program. International journal of rehabilitation research, 26(4), 329-333. (Wrong outcome)
2. Cook, S., Chambers, E., & Coleman, J. H. (2009). Occupational therapy for people with psychotic conditions in community settings: a pilot randomized controlled trial. Clinical Rehabilitation, 23(1), 40-52. (Wrong population)
3. Gehue, L. J., Crouse, J. J., Battisti, R. A., Yim, M., Carpenter, J. S., Scott, E. M., & Hickie, I. B. (2021). Piloting the ‘Youth Early-intervention Study’(‘YES’): Preliminary functional outcomes of a randomized controlled trial targeting social participation and physical well-being in young people with emerging mental disorders. Journal of Affective Disorders, 280, 180-188. (Wrong population)
4. Hamilton, A.B., Cohen, A.N., Glover, D.L., Whelan, F., Chemerinski, E., McNagny, K.P., Mullins, D., Reist, C., Schubert, M. & Young, A.S. (2013). Implementation of evidence‐based employment services in specialty mental health. Health services research, 48(6pt2), 2224-2244. (Wrong population)
5. Ikebuchi, E., Sato, S., Yamaguchi, S., Shimodaira, M., Taneda, A., Hatsuse, N., Watanabe, Y., Sakata, M., Satake, N., Nishio, M. & Ito, J.I. (2017). Does improvement of cognitive functioning by cognitive remediation therapy effect work outcomes in severe mental illness? A secondary analysis of a randomized controlled trial. Psychiatry and clinical neurosciences, 71(5), 301-308. (Wrong population)
6. Iwanaga, K., Wehman, P., Brooke, V., Avellone, L., & Taylor, J. (2021). Evaluating the effect of work incentives benefits counseling on employment outcomes of transition-age and young adult Supplemental Security Income recipients with intellectual disabilities: A case control study. Journal of Occupational Rehabilitation, 1-11. (Wrong population)
7. Kaehne, A. (2016). Project SEARCH UK–evaluating its employment outcomes. Journal of Applied Research in Intellectual Disabilities, 29(6), 519-530. (Wrong study design)
8. Kuipers, E., Holloway, F., Rabe-Hesketh, S., & Tennakoon, L. (2004). An RCT of early intervention in psychosis: Croydon Outreach and Assertive Support Team (COAST). Social psychiatry and psychiatric epidemiology, 39, 358-363. (Wrong outcome)
9. Linaker, C., Fraser, S., Price, C., Maguire, N., Little, P., Madan, I., Pinedo-Villanueva, R., Coggon, D., Cooper, C., Ntani, G. & Walker-Bone, K. (2021). Individualised placement and support programme for people unemployed because of chronic pain: a feasibility study and the InSTEP pilot RCT. Health Technology Assessment (Winchester, England), 25(5), p.1. (Wrong outcome)
10. Proudfoot, J., Guest, D., Carson, J., Dunn, G., & Gray, J. (1997). Effect of cognitive-behavioural training on job-finding among long-term unemployed people. The Lancet, 350(9071), 96-100. (Wrong population)
11. Reme, S. E., Grasdal, A. L., Løvvik, C., Lie, S. A., & Øverland, S. (2015). Work-focused cognitive–behavioural therapy and individual job support to increase work participation in common mental disorders: a randomised controlled multicentre trial. Occupational and environmental medicine. (Wrong population)
12. Twamley, E. W., Thomas, K. R., Burton, C. Z., Vella, L., Jeste, D. V., Heaton, R. K., & McGurk, S. R. (2019). Compensatory cognitive training for people with severe mental illnesses in supported employment: a randomized controlled trial. Schizophrenia research, 203, 41-48. (Wrong population)
13. Twamley, E. W., Vella, L., Burton, C. Z., Becker, D. R., Bell, M. D., & Jeste, D. V. (2012). The efficacy of supported employment for middle-aged and older people with schizophrenia. Schizophrenia research, 135(1-3), 100-104. (Wrong population)
14. Van Duin, D., De Winter, L., Kroon, H., Veling, W., & Van Weeghel, J. (2021). Effects of IPS plus cognitive remediation in early psychosis: 18-month functioning outcomes of a randomized controlled trial. Schizophrenia Research, 236, 115-122. (Wrong population)
15. Walker-Bone, K., Fraser, S.D., Price, C., Maguire, N., Cooper, C., Madan, I., Ntani, G. & Linaker, C.L. (2022). A pilot trial investigating the feasibility of a future randomised controlled trial of Individualised Placement and Support for people unemployed with chronic pain recruiting in primary care. Primary Health Care Research & Development, 23, e39. (Wrong outcome)
