# Supplementary material for: The effectiveness of interventions in reducing economic inactivity for people with long term health conditions and disabilities in the United Kingdom: a systematic review
Source: BMC Public Health. 2025 Dec 30;25:4400. doi: 10.1186/s12889-025-25708-3 (PMC12754864; doi:10.1186/s12889-025-25708-3)
Supplement: Supplementary file 4 — Supplementary Material 4. [file 12889_2025_25708_MOESM4_ESM.docx]

| **Supplementary Material 4: Summary table of included studies** | | | | | | | |
| --- | --- | --- | --- | --- | --- | --- | --- |
| **Study**  **Author and year**  **Reference(s)** | **Aim** | **Population** | **Intervention** | **Study design** | **Outcome measures** | **Results** | **Conclusions** |
| 1. Pathways to work  Adam et al 2006[36] | To evaluate the impact of Pathways to Work | 8035 incapacity benefits claimants in 7 pilot areas (Bridgend, Derbyshire, East Lancashire, Essex, Gateshead/South Tyneside, Renfrewshire, Somerset) | Compulsory work focused interviews with threat of sanctions for non-attenders, programmes to increase employability, financial incentives for employment | A ‘difference-in-differences’ approach comparing outcomes between those in pilot areas and those in matched comparison areas | Employment, earnings, incapacity benefit receipt, self-reported health status | At 42 weeks, the Pathways to Work cohort were more likely to be working (+9.4% p<0.001), report an increase in income (+£71.73, p<0.001) less likely to be in receipt of incapacity benefit (-8.2%, p<0.001) and less likely state a health problem affecting employment (-2.9%, p<0.05). Results did not differ by gender or age | Findings suggested statistically significant impacts of Pathways to Work on employment. Only new applicants were eligible limiting generalisability |
| 2. Pathways to work  Bewley et al 2007[37] | To evaluate the effect of Pathways to Work | 5784 incapacity benefits claimants in 7 pilot areas (Bridgend, Derbyshire, East Lancashire, Essex, Gateshead/South Tyneside, Renfrewshire, Somerset) | Compulsory work focused interviews with threat of sanctions for non-attenders, programmes to increase employability, financial incentives for employment | A ‘difference-in-differences’ approach comparing outcomes between those in pilot areas and those in matched comparison areas | Employment, earnings, incapacity benefits receipt, self-reported health status | Pathways to Work cohort were more likely to still be working a year and a half later (+7.4%, p=0.09). Conversely, there were no long-term improvements in income, receipt of incapacity benefit, or health problems affecting employment. Females (13%, p<0.05) and those who had children to support (17.6%, p<0.05) were more likely to be employed | Results suggested Pathways to Work continued to have a positive and sustained impact on employment. See Adams for issues of generalisability |
| 3. The EQOLISE trial  Burns et al 2007[38]  Burns & Catty 2008[39]  Burns et al 2009[40]  Catty et al 2008[41]  Kilian et al 2012[42]  Knapp et al 2013[43] | To evaluate the effectiveness of individual placement and support (IPS) compared to current career services | 312 community-based patients, aged 18-65 years, with severe mental health conditions, 12 months unemployed but wanting to find work. Six European Centres (Bulgaria, Germany, Italy, Netherlands, Switzerland, UK) | (1) prompt search for employment; (2) emphasis on working in the open job market; (3) based on individual’s employment choices; (4) continued employment progression; (5) advice regarding benefits; (6) no grounds for eligibility; (7) no time restrictions; (8) situated with mental health teams | Multi-site, stratified randomised controlled trial (RCT) | Number of days employed, number of hours worked, employment contract length, hospital admissions, quality of life, clinical functioning, social functioning, use of service | In the UK 48% of participants commenced employment over 18 months of assistance via IPS, compared to 16% in the control group of existing career services. IPS was significantly more effective than the existing career service in terms of career outcomes (difference 32.0%, 95% CI 7.7–56.3). There were, however, considerable differences in findings across the six European centres in terms of IPS effectiveness, thought to be accounted for by differences in labour market conditions in each area | Overall IPS was reported as being more cost effective, in terms of health and social care, than existing vocational services. Type of work secured through IPS typically low skilled / support roles even in favourable labour market conditions. Recruitment to the study was especially difficult in countries with a substantial benefit trap including the UK |
| 4. IPS LITE  Burns et al 2015[44] | To evaluate the effectiveness and cost effectiveness of time limited IPS | 123 community-based patients, aged 18-65 years, in contact with Oxfordshire Community Mental Health Team, 6 months unemployed but wanting to find work | (1) prompt search for employment; (2) emphasis on working in the open job market; (3) based on individual’s employment choices; (4) continued employment progression; (5) advice regarding benefits; (6) no grounds for eligibility; (7) time restrictions; (8) situated with mental health teams | RCT | Employment (1 day+), employment contract length, number of days employed, clinical functioning, social functioning, costs associated with service use | 41% IPS-LITE patients found work after a year and a half compared to 46% standard IPS although 97% of IPS-LITE patients had been released from the IPS-LITE service compared to only 28% from standard IPS. At 40 weeks only 6% of IPS-LITE patients and 11% of standard IPS patients were in work. There were also no significant differences in social or clinical functioning, duration of work, days employed or length of time to first job | IPS and IPS-LITE were as effective as each other however, as IPS-LITE came to an end more quickly, more patients could make use of the service. Authors calculated that the extra capacity matched with the service outcomes could results in an additional 17% of patients finding work at 40 weeks compared to standard IPS. Therefore, suggesting IPS-LITE was cost effective than standard IPS |
| 5. IPS  Deloitte 2017[45] | To assess the economic impact of IPS versus traditional vocational schemes | 126 individuals with severe mental health conditions referred by Community Mental Health Teams (CMHT) using an IPS service in Glasgow (n=41 secured employment) | (1) prompt search for employment; (2) emphasis on working in the open job market; (3) based on individual’s employment choices; (4) continued employment progression; (5) advice regarding benefits; (6) no grounds for eligibility; (7) no time restrictions; (8) situated with mental health teams | Economic Impact Assessment | Economic impact for service users, Scottish government and NHS | IPS had an estimated net economic impact of £180,970 for the year 2016 or £1,400 per user | Because more users secured employment via IPS and it was cheaper than alternative traditional vocational schemes it had a positive economic impact. Based on several assumptions from the literature |
| 6. ONE Advisory Service  Green et al 2003[46] | To assess the feasibility and effectiveness of delivering ONE via different delivery models | 4785 sick or disabled clients claiming disability related benefits in 12 pilot areas (Buckinghamshire, Calderdale & Kirklees, Clyde Coast, Essex SE, Lea Roding (N.E. London), Leeds, North Cheshire, North Nottinghamshire, Somerset, Southeast Gwent, Suffolk, Warwickshire) | Compulsory work focused interviews with Personal Advisor with threat of sanctions for non-attenders. Delivered via a Basic model, a Call Centre model and a Private/Voluntary Sector model | Controlled study comparing outcomes between those in pilot areas and those in control areas | Employment (of more than 2 days per week) | Rates of employment increased for both those accessing the ONE advisory service (24% to 28%) and the controlled comparison group (20% to 25%) - not a statistically significant difference. Significant differences by delivery model with the Basic model outperforming the Call Centre and Private/Voluntary Sector models | ONE not effective at increasing rates of employment |
| 7. SWAN (Supported Work and Needs) study  Howard et al 2010[47]  Heslin et al 2011[48] | To assess effectiveness and cost-effectiveness of IPS | 219 community-based patients with severe mental health conditions, 3 months unemployed but wanting to find work, in two distinct areas of London | (1) prompt search for employment; (2) emphasis on working in the open job market; (3) based on individual’s employment choices; (4) continued employment progression; (5) advice regarding benefits; (6) no grounds for eligibility; (7) no time restrictions; (8) situated with mental health teams | RCT | Employment (minimum 1 month), service use, psychosocial functioning | No significant difference between groups after one year with only 13% of patients receiving IPS finding work compared to 7% of patients receiving existing services. No significant differences in period of employment, income or hours employed each week. A third of patients in the IPS group failed to engage with the employment advisor. The average number of sessions undertaken with the employment advisor was 14. 86% of patients followed up at 24 months, but few were working in the competitive labour market across both arms. Significantly more patients (P=0.041) were employed in the IPS arm (22%) compared to the control arm (11%). Costs did not significantly differ by arm | Study context of a socially deprived area and an intervention provided by an external agency may have made implementation more difficult. However, these unexpectedly low results are partly explained by a conservative definition of employment, a month or more in a job, rather than the usual 1 day or more of competitive employment |
| 8. ONE Advisory Service  Kirby & Riley 2003[49]  Kirby & Riley 2004[50] | To assess the effect of ONE via three delivery models (See Green et al. 2003). | 29451 sick or disabled clients claiming disability related benefits. 5% random sample of UK claimants of disability related benefits | Compulsory work focused interviews with Personal Advisor with threat of sanctions for non-attenders. Delivered via a Basic model, a Call Centre model and a Private/Voluntary Sector model | Difference in difference procedure | Employment, benefits status | No difference in the receipt of benefits following the ONE advisory service compared to a comparison group. Claimants left benefits more quickly when involved in the earliest phases of the ONE Advisory Service compared to those involved in the later phases suggesting a decline in employment outcomes and a potential cohort effect | The evidence did not suggest that the ONE advisory service (via any of the models of delivery) was effective in increasing the numbers of people with long term health conditions and disabilities leaving benefits |
| 9. New Deal for Disabled People Personal Adviser Service  Loumidis et al 2001[51] | To evaluate the New Deal for Disabled People Personal Adviser Service | 2557 people in receipt of incapacity-related benefits for 7+ months in 12 pilot areas: Bedfordshire, Bolton, Bristol East and Bath, Central Sussex, Eastern Valleys, Lanarkshire, Newham, North Yorkshire, Sandwell, South Devon, South Tyneside, Spalding | The New Deal for Disabled People Personal Adviser Service aimed to assist people with a disability or health condition into work | Controlled study comparing participants with nonparticipants | Benefits status | 11% of those who participated in the Personal Advisor Service were no longer claiming benefits compared to 7% of those who did not participate over a 24 month follow up period. Those who participated in the Personal Advisor Service exited benefits more quickly than those who did not although the probability of exiting from benefits was higher for participants who had been claiming benefits for the shortest period of time (p<0.05) | There were no significant differences in exit from benefits between participants and non-participants of the Personal Advisor Service. Exit from benefit did not automatically translate into competitive employment |
| 10. IPS employment specialist  Marwaha et al 2014[52] | To assess the effectiveness of IPS provided by a skilled employment advisor (Model B) compared to IPS provided by existing staff who had received additional training to provide the intervention (Model A) | 106 community-based patients with severe mental health conditions accessing 5 mental health teams in the West Midlands | (1) prompt search for employment; (2) emphasis on working in the open job market; (3) based on individual’s employment choices; (4) continued employment progression; (5) advice regarding benefits; (6) no grounds for eligibility; (7) no time restrictions; (8) situated with mental health teams. Either provided by skilled advisor or existing staff | Natural experiment | Employment, demographics, health status, benefits status | Jobs in the open labour market were secured by 10.3% patients receiving Model A IPS compared to 22.8% patients receiving Model B IPS and 17.7% of all patients in receipt of IPS. These rates rose to 25.6% for Model A and 35.1% for Model B when patients receiving training were also included however none of these differences were significant | Authors concluded that it was possible to implement IPS into existing UK mental health teams to increase rates of competitive employment in a timely manner. Despite the study showing no significant difference in the method of IPS service delivery on rates of competitive employment authors concluded that employing a specialist employment advisor was more effective in securing jobs for patients compared to training existing members of staff to provide IPS |
| 11. Health-led Employment  Newton & Gloster 2023[53]  Dorsett et al 2023[54]  Edney et al 2023[55]  Newton et al 2023[56] | To assess the effectiveness of IPS | 7266 community-based patients with long term health conditions and disabilities who were economically inactive in West Midlands Combined Authority (WMCA) and Sheffield City Region (SCR) | (1) prompt search for employment; (2) emphasis on working in the open job market; (3) based on individual’s employment choices; (4) continued employment progression; (5) advice regarding benefits; (6) no grounds for eligibility; (7) time restrictions; (8) situated with mental health teams | RCT | Employment (3+ months), earnings, health (EQ5D5L) and wellbeing (SWEMWBS) | In WMCA, IPS-LITE had a significant effect on the likelihood of working for more than three months across the 12 months after being randomised (4 percentage points at the 99% significance level) with the IPS-LITE group being 20% more likely, than the control group, to have worked for more than three months after being randomised. While there were no effects on health and wellbeing in the WMCA IPS-LITE group, there were improvements in the SCR IPS-LITE group. When both WMCA and SCR groups were pooled there was no evidence of an employment or earnings effect of IPS-LITE but there were small effects on health and wellbeing both statistically significant at the 95% level. A cost benefit analysis, based on the effects of IPS-LITE on health and wellbeing outcomes, reported a return on investment for IPS-LITE of £1.22 for every £1 spent across both WMCA and SCR groups | Results of the Health-led Employment RCT were influenced by the authors choice of primary outcome measure (working for more than three months) which, while being more realistic of true employment status was more challenging to achieve than other similar studies |
| 12. New Deal for Disabled People (NDDP)  Orr et al 2007[57] | To evaluate the impact of the New Deal for Disabled People | 566645 people claiming disability-related benefits in the UK who were eligible for the New Deal for Disabled People | All disability-related benefit claimants were invited to take part in an interview with a job brokers about employment on a voluntary basis. This triggered access to other work focussed opportunities including training, personalised work searching activities, job advice and ongoing support once employed | Controlled study comparing registrants with non-registrants | Employment, benefit status | After two years there were significant (p<0.05) increases in rates of competitive employment for both new (+7%) and current (+11%) New Deal for Disabled People registered benefit claimants particularly for long-term/high-rate claimants and those facing multiple or significant barriers to employment. Over two years there were also significant (p<0.001) exits from benefits for both new (-13%) and current (-16%) New Deal for Disabled People registered benefit claimants particularly for older, more disabled long-term claimants and those facing multiple or significant barriers to employment | Participation in the New Deal for Disabled People was voluntary and as such the intervention sample for the study was not random which could have introduced selection bias as volunteers may have been more motivated to find work |
| 13. Steps2Wellbeing IAPT  Purdon & Bryson 2022[58] | To evaluate Employment Advisers (EAs) in Improving Access to Psychological Therapies (IAPT) | 6640 IAPT clients who had long term health conditions and disabilities or were carers, unemployed or stayed-at-home in Southampton and Dorset | Voluntary, evidence-based guidance for treatment of people with depression, anxiety, and other frequently occurring psychological problems designed to enhance provision of psychological therapies combined with support and advice regarding employment | Controlled study comparing IAPT clients who saw an EA (11%) with a matched comparison group of IAPT clients who did not | Employment, benefit status, mental health status, daily functioning | By the last appointment, IAPT clients who were unemployed at baseline and who saw an EA had significantly better mental health, were significantly less likely to believe that their mental health influenced their prospects of employment, were significantly less likely to be in receipt of disability related benefits, and were significantly more likely to be employed than the matched sample who had not seen an EA. EAs also supported participants in identifying and claiming benefits for which they were eligible but were not claiming at baseline. EAs in IAPT did not have any effect on participants self-reported everyday activities | Seeing an EA in IAPT had a significant impact on mental health and on the likelihood of entering the labour market |
| 14. IPS  Rinaldi & Perkins 2007[59]  Rinaldi & Perkins 2007[60] | To evaluate the effectiveness of IPS provided by an employment specialist compared to non-integrated pre-vocational services | 451 community-based patients with severe mental health conditions in 12 mental health teams in Kingston, Merton and Sutton | (1) prompt search for employment; (2) emphasis on working in the open job market; (3) based on individual’s employment choices; (4) continued employment progression; (5) advice regarding benefits; (6) no grounds for eligibility; (7) no time restrictions; (8) situated with mental health teams. Either provided by employment specialist | Natural experiment | Employment | The IPS specialist employment advisors supported 38% patients into employment or training compared to 12% in the non-integrated pre-vocational services (p<0.001) at 6 months. At 12 months the IPS specialist employment advisors supported 39% patients compared to 10% in the non-integrated pre-vocational services (p<0.001). Cost of supporting a patient into competitive employment in the non-integrated pre-vocational service was 6.7 times higher than in the IPS service | Authors concluded that the results not only corroborate the implementation of IPS in community mental health teams but also indicate the importance of specialist employment advisors in providing the service |
| 15. Work-focused counselling (IPS enhanced with work-focused CBT)  Schneider et al 2016[61] | To examine the effectiveness of IPS plus Cognitive Behavioural Therapy (CBT) compared to IPS | 74 community-based patients, aged 18-60 years, with severe mental health conditions, unemployed but wanting to find work from Nottinghamshire Healthcare National Health Service (NHS) Trust | (1) prompt search for employment; (2) emphasis on working in the open job market; (3) based on individual’s employment choices; (4) continued employment progression; (5) advice regarding benefits; (6) no grounds for eligibility; (7) no time restrictions; (8) situated with mental health teams. Plus 3-6 sessions of CBT | Pilot RCT | Employment (number of hours per week), training, education, volunteering, costs | A third of participants secured competitive employment. No significant difference between participants receiving CBT+IPS and participants receiving IPS in terms of mean hours per week (hpw) employed at either six month (CBT+IPS 3.89hpw (SD 10.60) v IPS 3.22hpw (SD 9.53)) or 12 month (CBT+IPS 7.07hpw (SD 14.09) v IPS 3.67hpw (SD 7.80)) follow up | Over the course of the study 43% of participants withdrew, particularly those who were not engaging with the services, affecting the power of the study to determine effectiveness of CBT+IPS over IPS alone |
| 16. IPS  Van Veggel et al 2015[62] | To examine the effective of IPS | 247 patients with severe mental health conditions, in Sussex | (1) prompt search for employment; (2) emphasis on working in the open job market; (3) based on individual’s employment choices; (4) continued employment progression; (5) advice regarding benefits; (6) no grounds for eligibility; (7) no time restrictions; (8) situated with mental health teams | Between group study with 3 services collecting data before and after implementation of IPS | Employment | IPS led to significantly more patients (24.9%) working in the open labour market compared to before IPS (14.3%). IPS also resulted in employment of a greater number of hours per week with 24.3 hours/week compared to 15.4 hours/week and a shorter time to securing employment with 153 days to employment compared to 371 days. | Authors note however, a vulnerability of IPS to loss of effect size over wider reaching implementation due to issues attaining required levels of work in a competitive labour market and maintaining fidelity of intervention. |
